# Rapid and Responsive Record Linkage to support NSW Health COVID-19 pandemic response.

**DOI:** 10.23889/ijpds.v7i3.1960

**Published:** 2022-08-25

**Authors:** Michael Nelson, Victoria Pye, Pooja Nair, Tim Harrold, Lisa McCallum, Lee Taylor

**Affiliations:** 1 NSW Ministry of Health

## Objectives

To support the NSW Health COVID-19 pandemic response using rapid record linkage to provide key information to inform policy and practice.

## Approach

The Centre for Health Record Linkage hosts a secure, high-performing data linkage system, including a Master Linkage Key (MLK) of administrative health datasets, and generates linked data to inform policy decisions.

Over the course of the COVID-19 pandemic, a series of rapid record linkage projects were requested for a range of purposes.

1. Characteristics of people hospitalised with COVID-19
2. Characteristics of the NSW Health workforce notified with COVID-19
3. Information on COVID-19 tests carried out on Aboriginal people and people born overseas
4. COVID-19 Vaccine Effectiveness in the Sydney Metropolitan area

## Results

The COVID-19 Communicable Disease Register – a linked dataset of COVID Case notifications, negative COVID tests, Admitted Patient Data, Emergency Department Data, Death Registrations and records of the Australian Immunisation Register, has been updated 34 times since March 2020, providing a rich dataset to answer questions on the health outcomes of COVID-19 cases, such as hospitalisations and ICU admissions, on access to testing in vulnerable populations, and on vaccine effectiveness in the Sydney Metropolitan area. The update frequency, scope and size of the Register has changed throughout the pandemic. We will describe examples of how this information has been used to inform policy decisions at different points in the pandemic response.

## Conclusion

The combination of an up-to-date MLK, established data supply infrastructure and procedures, and supportive legislation enabled NSW Health to provide rapid and responsive record linkage to support the COVID-19 pandemic response.

